# Experiences of patients with endometriosis with a digital health application: a qualitative analysis

**DOI:** 10.1007/s00404-024-07651-7

**Published:** 2024-07-27

**Authors:** Marco Richard Zugaj, Ariane Germeyer, Karina Kranz, Andrea Züger, Jens Keßler

**Affiliations:** 1 Medical Faculty Heidelberg, Department of Anaesthesiology, Pain Medicine Section, Heidelberg University, Heidelberg, Germany; 2 Medical Faculty Heidelberg, Heidelberg Women’s Clinic, Department of Gynaecological Endocrinology and Fertility Disorders, Heidelberg University, Heidelberg, Germany; 3 Medical Faculty Heidelberg, Center for Psychosocial Medicine, Institute for Medical Psychology, Heidelberg University, Heidelberg, Germany; 4 Institute for History, Theory and Ethics of Medicine, Justus Liebig University Giessen, Giessen, Germany

**Keywords:** Semi-structured interview, Chronic pain, Endometriosis, Healthcare app, Quality of life, Women’s health

## Abstract

**Background:**

Endometriosis is a frequent disease in women of reproductive age in which the endometrium occurs outside the uterine cavity. Multimodal treatment approaches are necessary due to loss of quality of life and the chronic nature of the disease. Digital health applications (DiGa) are becoming increasingly important. This research project investigates how a healthcare app can influence the subjective experience of illness in patients with endometriosis.

**Methods:**

Empiric data were collected through semi-structured interviews. Data analysis was carried out using qualitative focussed interview analysis. Reliability was ensured by joint interdisciplinary and interprofessional evaluation of the interviews by experts and those affected.

**Results:**

Ten patients with endometriosis and the prescribed healthcare app Endo-App^©^ were examined. Categories were defined from the superordinate categories “Factors influencing the experience of illness” and “Evaluation of the app”. The app provided reliable information, promoted self-efficacy through exercises and strengthened the perception of the individuality of the illness. It helped to minimise nocebo effects from internet research and enabled a positive change of perspective. Patients criticised the time required for data input and had data protection concerns. The educational elements were often seen as redundant. Some patients only used the app briefly, or not at all.

**Conclusion:**

Once a DiGa has been prescribed, it may be useful to explain its use on an outpatient basis and validate regular use. Blind re-prescribing of DiGas should be avoided. Younger patients with a recent diagnosis or patients following rehabilitation may benefit more from prescribing.

**Supplementary Information:**

The online version supplementary material available at 10.1007/s00404-024-07651-7.

## Introduction

Endometriosis is defined as the spreading of functional endometrium to any location outside the uterine cavity [1]. Endometriosis is one of the most common diseases in women of reproductive age [2]. Endometriosis-related hospital admissions have risen continuously in recent years [3]. Qualitative data show a high level of suffering among patients [4]. In addition to the main symptoms of pain and infertility, there are more subtle symptoms [5]. Women with endometriosis suffer from a reduced quality of life, an increased incidence of depression, negative effects on intimate relationships, restriction or reduction of social activities, loss of income and an increased risk of other chronic diseases [3, 6]. Endometriosis patients cause considerable direct and indirect costs in the healthcare system [3, 7].

As endometriosis is a chronic disease, a long-term therapy concept is required. Conservative (medicinal/hormonal) forms of therapy and surgical measures form the basis [8]. A series of supportive and integrative measures are necessary to compensate for the secondary myofascial complaints of the patients as a result of the chronification mechanisms and to achieve a general symptom relief and disease management [9] [10–12]. In multimodal and interdisciplinary cooperation between pain therapists, gynaecologists, psychotherapists or psychosomatic specialists, nutritionists and physiotherapists, an increase in the quality of life for patients with endometriosis pain can be achieved [13–15].

Since the Digital Healthcare Act from December 2019, DiGa can be prescribed by German doctors and psychotherapists [16]. Health insurance companies reimburse the costs if the apps has been tested and approved by the Federal Institute for Drugs and Medical Devices (BfArM) [17, 18]. Germany continues to be an international pioneer in this area [16]. Studies on other chronic pain disorders have already shown that patients can benefit from an app supporting therapy [19–21]. The number of apps available has increased in recent years [22–24].

The healthcare app “Endo-App^©^” is now addressing German-speaking patients with endometriosis. The main features are an endo-diary, learning modules, interactive exercises, an endo plan based on the documented diary entries, evaluation based on intelligent valuation of entries, and the possibility to create a personal SOS-Plan. Following successful testing by the BfArM, the app can be prescribed by treating physicians at the expense of statutory health insurance companies [16, 25].

An objective assessment of DiGas is difficult, but is of growing importance from a health policy perspective [26, 27]. Industry independent evidence-generating research is necessary. Therefore, the aim of this research project was to independently identify how the illness experience and lifeworld reality of patients with endometriosis are influenced by a prescribed healthcare app.

## Materials and methods

In addition to quantitative methods such as questionnaire surveys, qualitative methods are playing an increasingly important role in researching multidimensional phenomena such as pain. The aim of these methods is to capture the patient’s internal perspective and to explore the subjective attribution of meaning with regard to their experience of illness. Qualitative methods make it possible to triangulate and contrast the results of quantitative research through a change of perspective and help to discover new research approaches. An endometriosis patient was part of the research team and involved in various steps in the research process (development of interview guide, data analyses, reporting and dissemination).

### Recruitment, inclusion criteria and sampling strategy

Patient recruitment took place between February and August 2023. Patients were recruited via a poster in the waiting area of the Pain Centre at Heidelberg University Hospital. Inclusion criteria were: Patients with endometriosis (ICD10: N80.0-9), prescription of the DiGa: “Endo-App^©^”. Exclusion criteria were lack of legal capacity to consent, age under 18 and insufficient knowledge of German language.

Sampling was carried out by the study director (MZ) using a deductive strategy (dependent on prior theoretical knowledge) based on entries in the patient records. To achieve the greatest possible variance and heterogeneity the predetermined criteria for sample selection were: all age groups; all dynamics of pain; varying disease stages; disturbed or undisturbed relationship with the practitioners. The sample should be large enough to achieve theoretical saturation and to find a sufficient number of contrasting cases.

### Data collection: semi-structured interviews

The patients were interviewed 4 weeks after the app was prescribed by their pain specialists. The semi-structured interview enables openness, structuring and specification at the same time [28]. The interview guide was developed on basis of actual specialist literature [29], the research question and discussions within the research team (Online Resource 1). MZ (male, anaesthesiologist and pain therapist, experienced in qualitative pain research) conducted all interviews. For counteract patients’ social desirability, there was no current treatment relationship between the interviewer and the study participants.

### Data preparation

During the interview, the spoken word was recorded with a digital recorder (Philips DPM6700 complete set for author and assistant, Philips GmbH Market DACH Hamburg) and after that transcribed verbatim by a member of the research group (Philips dictation and playback software SpeechExec 10, Philips GmbH Market DACH Hamburg). The transcription of the spoken word was carried out consistently according to the transcription rules [30]. Data protection, pseudonymisation and anonymisation of the raw data were carried out in accordance with an audited data protection plan [31].

### Data analysis

The interview transcripts were analysed using a qualitative six-step content-structuring method according to Kuckartz and Rädiker [30, 32] (Table 1). The analysis was conducted with a data analysis software (MAXQDA Analytics Pro Training, VERBI Software, Berlin) and was carried out between July 2023 and September 2023. The analysis considered both inductive (newly generated from the material) and deductive (based on previous theoretical knowledge) approaches. This combination united openness and theoretical orientation and thus enabled broad access to the research interest. The aim was to achieve a holistic investigation of lifeworld phenomena by analysing individual cases [32].

**Table 1:** Detailed illustration of the six-stage process for a focussed interview analysis of empirical data according to Kuckartz and Rädiker (Kuckartz and Rädiker 2022)

Step 1: Data preparation and exploration	This step involved intensive reading of the interviews and writing initial summaries and text memos
Step 2: Deductive preliminary categorisation	The first categories were formed deductively from the guidelines and the prior theoretical knowledge of the researchers
Step 3: Basic coding	The preliminary categories were used to analyse the interviews and sections of text were assigned (coded) to the categories. Further categories were defined and finally anchored in a fixed category system
Step 4: Fine coding	A tabular listing of all text passages in a category allowed for a more in-depth analysis and differentiation into subcategories. Summaries were written for selected text passages to further emphasise them
Step 5: Data analysis	Data of interest to the research question was selected and contrasted
Step 6: Documentation	Documentation of all steps was checked and a research report was written. Data archiving was performed in accordance with the data protection concept

### Reliability of the data analysis

Reliability was established by the incorporation of multiple interdisciplinary perspectives. All interviews were double-coded. Furthermore, patient involvement in the data analyses was performed. The authors MZ and AG (female, senior consultant in gynaecological endocrinology and reproductive medicine, endometriosis expert, no previous experience with qualitative research) jointly evaluated interviews 1–5. A system of categories was established by consensus and differentiated in a second step. The authors MZ and KK (female, Master’s student in psychology, affected endometriosis patient, experienced in qualitative data analysis) analysed interviews 6–8. The existing category system was agreed upon, further differentiated and the category definitions narrowed down. The authors MZ and AZ (female, PhD in cultural studies, expert in qualitative social research) analysed interviews 9 and 10 together. Finally, the established category system was jointly evaluated. The empirical data were coded according to predefined coding rules [30]. Quality criteria for categories were predefined and consistently applied [30]. A step-by-step audit trail was created so that reviewers could follow individual phases of study planning, data acquisition and data analysis.

### Manuscript preparation and translation of the empirical data

The manuscript was written in accordance with the “Standards for Reporting Qualitative Research (SRQR)” guidelines [33]. By transcribing recorded qualitative data, information that is conveyed in the intonation or facial expressions and gestures of the speaker can be lost. Additionally, in translation phrases and implications that are recognisable to native speakers can be lost. The authors made a special effort to translate the research report and the supporting empirical data into English with the subtext largely preserved, using Artificial Intelligence (DeepL, DeepL SE, Cologne, Germany) for this purpose. A final cross-check followed by native-speakers. After using this service, the authors reviewed and edited the content. The authors therefore assume sole responsibility for the content of the publication.

### Ethics vote and registration

The Ethics Committee of the Medical Faculty of Heidelberg approved the study (S-610/2022). The study was registered prospectively (DRKS00030338).

## Results

### Patient characteristics

10 patients with an ICD-10 diagnosis of endometriosis were included 4 weeks after receiving a novel DiGa prescribed by their pain therapist and funded by their health insurance. A heterogeneous sample was selected (Table 2). The age of study participants ranged from 16 to 40 years. Disease burdens of the patients ranged from symptom-free under hormone therapy to severe restriction due to permanent and generalised pain with complex comorbidities.

**Table 2:** Summary of patient characteristics

Interview	Self-reported gender	Ethnicity	Age (years)	Initial diagnosis of endometriosis	Stage	Abdominal operations	Hormonal therapy	Concomitant diseases
1	Female	Caucasian	26–30	2021	ASRM I, ENZIAN 0	Laparoscopy 2021	Current desire to have children	Chronic pain syndrome Migraine without aura Depressive episodes
2	Female	Caucasian	26–30	2014	ASRM I-II ENZIAN 0	Appendectomy 2007, Laparoscopy for endometriosis 2014, 2019 and 2022; Caesarean Sect. 2021	Dienogest	Chronic pain syndrome Lumbar radiculopathy
3	Female	Caucasian	26–30	2020	ASRM II ENZIAN 0	Laparoscopy for appendectomy and endometriosis treatment 2020	Drospirenone mono in the long cycle (off-label)	Chronic pain syndrome Tension headaches Lumbar radiculopathy Obesity Renal cysts Chronic gastritis Depressive episodes
4	Female	Caucasian	26–30	2022	ASRM I ENZIAN 0	Laparoscopy of bland ovarian cyst in 2009 and 2016, laparoscopy of endometriosis in 2022, appendectomy and bland ovarian cyst in 2013	Desogestrel	Bronchial asthma Neurodermatitis
5	Female	Caucasian	26–30	2020	ASRM IV ENZIAN FA	Laparoscopic appendectomy 02/2020, Laparoscopic adhesiolysis and endometriosis cyst extirpation 11/2020	Dienogest	Chronic pain syndrome
6	Female	Caucasian	36–40	2018	–	Explorative laparoscopy as a child, Laparoscopy 2018, Laparoscopy 2019	Currently not desired due to side effect	Budd-Chiari syndrome Meulengracht’s disease Chronic pain syndrome Migraine without aura Lumboischialgia Moderate depressive episode Intermittent porphyria
7	Female	Caucasian	21–25	2021	ASRM IV ENZIAN B2	Laparoskopy for endometriosis cyst extirpation and endometriosis repair 2021	Ethinylestradiol plus levonorgestrel in the long cycle (off-label)	Migraine with aura as a teenager Psoriasis arthropathy
8	Female	Caucasian	26–30	2011	Progress from ASRM I, ENZIAN FO (2020) to ASRM IV ENZIAN FO (2022)	Laparoscopy for endometriosis treatment 2011, 2014, 2016, 2018, 2020, 2022 (plus adhesiolysis)	Currently not desired due to side effects	May-Thurner syndrome Lumbosacralgia
9	Female	Caucasian	26–30	MRT 2022	#Enzian PxO0T3A1FI (MRT)	–	Current desire to have children	–
10	Female	Caucasian	16–20	Suspected diagnosis since 2020	–	Not yet desired	Ethinylestradiol plus dienogest	–

Over 5 h of interview material was transcribed and analysed. 65 categories were developed and allocated under the two main categories “Factors influencing the experience of illness” and “Evaluation of the app“(Online Resource 2)”. 744 segments were assigned to the categories. 58 memos with ideas and paraphrases were created for the text.

**Fig. 1:**
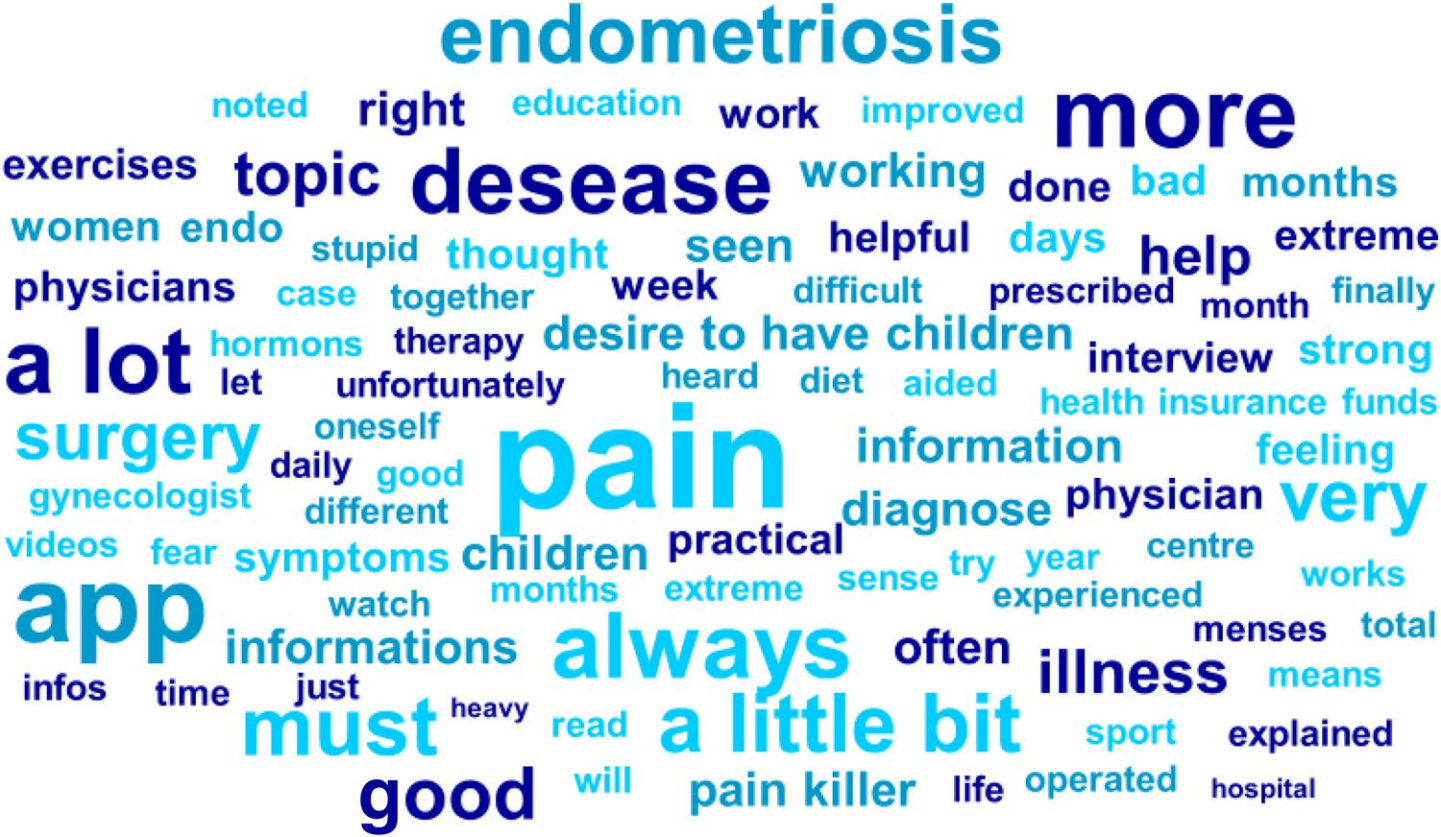
Word cloud with the words most frequently mentioned by the participants

A word cloud was created to visualise the most frequent words (Fig. 1). Pronouns, articles and filler words have been removed. It was found that terms such as “pain”, “endometriosis”, “always”, “surgery”, “desire to have children” and “app” were used particularly frequently.

### Factors influencing the experience of illness

#### Information gathering and nocebo

Patients expressed that reading a condensed summary of information about their condition through a prescribed app, covered by their health insurance, instilled confidence in the provided information:

B: Well, because it’s a specially developed app for endometriosis, it’s also trustworthy. That means you can trust what you read and on the internet it was always like that at the beginning—well, you just read through it, but is it really all true?

In contrast to this, research on the internet sometimes revealed disturbing information that led to uncertainty and fears:

B: Well, when I did some research on the internet, I must say I was very shocked. I was also frightened by all the things I read about how this could manifest itself, that it might not be possible to fulfil my desire to have children. That was the worst thing for me.

B: I remember the very first piece of information very well. It was in hospital, because the day after the operation the doctor came in and said: “Do you already know what you have? Has anyone told you yet?” And then I said: “No. Nobody was there.” And he said: “Yes, it’s endometriosis.” Then I asked him what it was and the doctor said to me: “Just google it.” And that’s what I did. And that was my first piece of information about endometriosis. As expected, it was just horrendous. Because you can find an incredible amount of information on the internet about women who have had lots of operations, who have had their organs affected and who have had something removed. And I think that was just terrifying.

#### Recognising the individuality of the disease

Our patients realised that it is not possible to make any predictions about the course of their own illness based on the progression of other patients. The app supported recognising the individuality of the disease’s course:

B: Because many patients have endometriosis and it’s different for everyone, but what about me and what information is relevant for me.

B: […] because I have experienced this all too often in therapy, that it always boils down to this. Wanting children, children, children, children. And I thought it was really good that the app doesn’t do that.

#### Promoting self-efficacy

Participants rated self-efficacy-enhancing education and guided physical exercises positively. Self-efficacy in coping with the illness was strengthened. Individually, using the app could have a positive impact on the perception of pain:

B: I know that if I have this episode now, for example, when I’m not feeling well at all and I’m at home, at least I know what I can do during that time. I can do yoga, relaxation exercises, but I can also make sure that I don’t let myself go during this time.

#### Association and framing of the disease

Patients reported that the educational programs provided by the application affected their personal perception and led to a shift in how they framed their illness. In some cases, an increase in acceptance of the illness was observed. A transition from an anatomical-pathological understanding of the disease to a physiological-dynamic understanding was achieved. In addition, acceptance of a bio-psycho-social disease model and a multimodal therapy approach was achieved:

I: And would you say that your perception of the disease has changed as a result of using the app? B: A little, yes. I: And how? B: I no longer see the illness as such a burden for me. It also explains to me how I can deal with the condition and what could help me to reduce the pain.

B: And I think you also have to work a lot on yourself to come to terms with the illness and rework and rethink your own structures.

#### “To be recognized”

A common narrative pattern is the lack of social esteem and attention for endometriosis. A lack of “being recognized” in a social context is reported by all patients. Therefore, Patients positively rated the exclusive addressing of the disease endometriosis through the application:

B: Well, you’re actually ill and therefore need protection, including in the work context, for example protection against being let go. It’s incredibly difficult because the illness is not recognised.

B: I was prescribed the app. I was really pleased about it because I thought it would be nice to finally have something for endometriosis to support it a little.

### Evaluation of the app

The application provides relaxation exercises through video tutorials, which has frequently received positive evaluations:

B: What helps me a lot are the relaxation exercises. I notice that very positively. When I’m stressed and I do one of these exercises, it helps me to calm down a lot.

B: But the individual relaxation exercises, for example, […] I really like that. Just coming down and forgetting about everyday life.

One patient did not like the way the guided meditations were spoken:

B: The narrator had such a compassionate voice I was in tears. And I had to stop at that point because I felt so sorry for myself […].

Guided physiotherapy measures were often rated as helpful and interesting:

B: I’ve never heard that before either. For me, the pelvic floor is when you’re pregnant. But I hadn’t read anything on the internet about doing it this way before.

Other tutorials provide education on endometriosis, the physiology of pain development and pain assessment. Some of the interviewed patients responded positively to the educational content.

B: And then to be able to read this information page again. That helped a lot and even today, if something comes up, I have a look at it first.

B: I imagine that if I’d had the diagnosis back then and had had this app, it would have helped me a lot.

Some patients stated that they had not gained any additional knowledge from the offered education:

B: […] because I had already done a lot of reading through a book, […] there wasn’t much new for me personally.

The pain diary offered was evaluated ambivalently. The possibility of objectifying the burden of illness and being able to monitor changes longitudinally was positive.

B: Well, first of all, I thought the structure of the app was great. In addition to the various exercises, there is also a calendar, which helped me a lot, especially because my gynaecologist or the women’s clinic wanted me to keep a diary of my pain.

B: And (.) just to know that you can get an overview of how much it actually takes up and not always purely from an emotional point of view. But really how much is it in reality? And it definitely helped me to get real insights into that.

The time required (hurdle), the daily preoccupation with disease symptoms (fixation) and the excessive demands of the selection options in the pain diary were negative:

B: Entering the symptoms into the app, for example, is hell for me.

B: I did that for a while with the pain diary […] But I also realised it wasn’t good for me to deal with the pain and think about it all the time. What were they like and when were they worse? My whole life revolves around the illness and the pain anyway.

Dietary changes were rarely implemented, but the nutritional information was new to many patients:

B: Well, I didn’t find out anything about nutrition on the internet and from friends and gynaecologists, for example. […] That helped me a lot, even though I eat the same diet anyway. That’s not a big change.

#### User behaviour

Clear patterns of use emerged. One patient could not use the app because of time constraints. Some used it intensively at first, but gradually reduced their use until they stopped:

B: […] I used it euphorically on the first, second and fourth day because I said I wanted to test it, because you have to be able to do something with it. But then it was actually far too much for me. […] It simply cost me an incredible amount of time. […] So, as I said, because I have four children, it was simply an enormous amount of time for me.

B: It was just too much for me personally, as I said before. And then I started to let it creep up on me. Then at some point I—well, you can be reminded that you should or can use this app. At some point, I switched it off and then I stopped using it and sort of let it fade out.

B: (laughs) Well, I really haven’t used it at all in the last few months. At some point I stopped looking at it and then I deleted the app after the code expired.

Some patients stated that they did not use the app regularly, but when necessary if they were feeling unwell:

B: Well, if you’re doing well, then you don’t tend to look at it and if you have some kind of complaint and then think oh, what could I do for myself now?

B: Because, of course, there are also lots of exercises that take, I don’t know, maybe half an hour or longer and you can’t always fit them into your daily routine. So, I didn’t use the app every day either. So I don’t think anyone will probably do that either. It’s probably too much at some point.

Two patients stated that they were already able to implement all measures on their own so that they could act independently of the app:

B. So I also ended up doing a few yoga exercises where I didn’t need my mobile phone at all. Where I knew okay hey, I can do it like this. I don’t have to use the app forever. But they were definitely a good part of the app, especially in this learning process of how to use it.

Two patients used the app regularly, even for months.

B: I was prescribed the app. I think 2 months ago. I can’t remember the last time I was there. And I’ve been using it every day since then.

B: Exactly, I’ve already had the second activation code sent to me. I’ve already entered it because I’ve definitely said that it’s incredibly useful for me. Especially when the diagnosis was just not that long ago, so if I’d had it two and a half years ago, I think it would have made things a lot easier.

#### Options for improving the app

In addition to the time required to use the app correctly and regularly, patients described other shortcomings. One patient had concerns about data security and the use of personal data:

B: After you have logged in, another authentication procedure pops up, for example with a fingerprint and the information that the data will also be passed on by the operating system, etc., which is too insecure for me personally.

Three patients had a negative view of incentives and automated reminders, believing that they sometimes put extra pressure on patients:

B: […] on the other hand, if you haven’t used the app for two or three days in a row, you get bombarded with emails. “Is everything OK with you?” Or you get an email saying: “Hello?” And then you think to yourself, that you’re simply having two days without pain.

The exchange with other patients, from which one patient had subjectively benefited, was missing in the app:

B: I’ve heard a lot from other women on social media, especially on Facebook, about how they deal with the disease. I miss that a bit in the app, this exchange with others. Of course, not everything works for every woman, but I miss having this support.

Specific suggestions for improvement were to integrate information about concomitant diseases of endometriosis and to offer help in applying for welfare state support:

B: […] there are so many concomitant illnesses that you hear about again and again, if there was perhaps a bit of an overview. Because that also helped me back then. […] for example, that I have a lactose intolerance. […] hypothyroidism, or that many people have fibromyalgia.

B: So maybe a bit more help in the Endo App with the degree of severe disability […] how to apply for it.

#### Wishes for the future

In expressing their expectations for the future, patients voiced specific concerns, addressing all stakeholders in the healthcare system. Their primary focus was on the desire for enhanced care, coupled with a more effective reduction in the burden of disease:

B: So for me, I hope that I no longer have the pain that I had.

B: Simply create this awareness first. First of all, hey, this disease exists, many people don’t know about it.

B: The only thing that really bothers me is the cost of fertility treatment. The fact that a disease has been proven, that problems can occur and then those affected still have to pay half of the costs if they are married. People are often pressurised into getting married.

B: Politicians could also do more to ensure that the disease receives more funding to advance research, and politicians also have a lot of influence on health insurance companies.

B: And also with the disability classification. I am at 50 per cent. I fought for it for a long time. I’ve always applied for a deterioration after an operation. [The degree of disability is determined by the German welfare office and may be relevant for other benefits, such as the provision of social services.]

## Discussion

The aim of this study was to describe patients’ experiences with a DiGa. A qualitative study approach in the sense of a focussed interview analysis according to Kuckartz and Rädiker was chosen. 10 patient interviews with over 5 h of interview transcripts were analysed. The analysis of the empirical data yielded main categories from two subject areas: “Factors influencing the experience of illness” and “Evaluation of the app”.

### Researcher and participant profiles

Forming an interdisciplinary and interprofessional scientific team, and ensuring equal and consensus-based evaluation of empirical data, facilitated a diverse approach to the research topic. The team comprises individuals from both natural science and humanities backgrounds, occupying various hierarchical positions and spanning a diverse age range. As a strength of our methodology [29], the findings were also reviewed by an expert affected herself.

The deductively selected patient group mainly represented severely affected patients with comorbidities. Various social milieus and various representative life stages were selected. Similar to other qualitative studies, patients with a high disease burden were more likely to participate [4]. Asymptomatic patients with an “incidental finding” of endometriosis could not be recruited. The motivation of this patient group to participate in studies is possibly lower than the motivation of severely affected patients and they may not be addressed by the recruitment modality. Interestingly the disease’s burden did not correlate with the disease’s stadium.

### Factors influencing the experience of illness

Endometriosis’ impact on women’s quality of live is high [5, 34–37]. Patients’ perspective on Endometriosis could be revealed in qualitative studies in the past [4]. Confirming this, we found several bio-psycho-social factors of the disease experience in our patient collective. In addition, we were able to identify a subjective influence through the use of an endometriosis DiGa.

It is known that pain diaries can record the subjective and affectively assessed pain experience more objectively. However, the pain diary also leads to a constant preoccupation with the pain, which can have a tiring effect [38]. Our patients confirmed these two effects.

It was striking that dyspareunia was rarely reported by our patients. This is analogous to the results of a systematic review by Facchin et al., which showed an avoidance of sexuality as a topic in doctor-patient consultations with endometriosis sufferers [39]. It is possible that the gender (male) of the interviewer contributed to the avoidance of the topic of sexuality in our interview study. However, it is also possible that the topic of sexuality is underrepresented in the app, so that the patients were unable to report any influence.

Self-efficacy, options for symptom control and recognising the individuality of one’s own disease progression can positively influence chronic pain experience [13] and are addressed in the app. A subjective positive effect was reported by our patients.

Risk factors for pain chronification such as catastrophising, state anxiety and stress [13, 21] could be counteracted. Nocebo information on the internet and social media is also a risk factor for pain aggravation [40]. This could be avoided by using information provided by the app. However, some patients felt that the education and information elements of the app were redundant.

One problem is still the marginalisation of endometriosis by those not affected. Among other things, this can result in patients’ presenteeism or absenteeism [5]. One reported positive effect is that patients experience appreciation through the prescription of a special endometriosis app (being seen). Awareness of the disease is also increased among non-affected people through campaigns and media attention.

Contact with healthcare stakeholders is still described as inconsistent and sometimes ambivalent. Nocebo information in particular is also conveyed by professionals. The topic of fertility is particularly fraught with anxiety. One patient reported annual surgeries in connection with abdominal pain. This may be a case of overtreatment with corresponding negative consequences for the patients.

### Evaluation of the app

Quantitative empirical data analyses have shown positive effects on the experience of illness (EHP-5) after 4 weeks of using the Endo-App^©^, but these data are industry produced and only available on personal communication with the company. In our sample, 6 patients surveyed stated that they benefited from the app. Fears in particular could be reduced. However, some patients used the app only briefly or not at all after the prescription and did not benefit subjectively. This behaviour is known from other chronic pain conditions [38].

It may make sense to introduce the DiGa on an outpatient basis, for example by medical assistants, in order to increase understanding and thus achieve patients’ longer-term adherence. According to our data, usage behaviour should be monitored after prescribing a DiGa and further prescriptions should not be given blindly.

The cost-effectiveness of the Endo-App^©^ has not yet been analysed. The question of whether a systematic approach to the disease endometriosis via an app is superior to other methods (outpatient therapy, day-care therapy, inpatient therapy, rehab) still needs to be investigated. Young sufferers and newly diagnosed sufferers may benefit more from the app. It is possible that patients benefit particularly after rehabilitation if what they have learnt is consolidated through repetition in the app.

Ultimately, we were able to contrast and expand the still young field of research on DiGa. Further industry-independent user studies are necessary to verify statements about DiGa, analogous to prescription drugs.

### Limitations

- The number of cases in qualitative research is small and the sampling does not fulfil the criteria of randomisation. Therefore, unlike quantitative research, which works with large numbers of cases, qualitative research cannot claim generalisation in the form of statistical representativeness [30]. In these cases, however, generalisation takes the form of an empirically based theory or the recognition of patterns and not the determination of statistical significance [30]. We provided extensive information on the sample size and the selection of patients in the methods section.
- Due to the setting of the study in the rooms of the university hospital and the person conducting the interview, no assumption of unfamiliarity can be made. Adaptation of the participants’ statements due to social desirability cannot be completely ruled out.
- The interviews were characterised by fundamental openness and non-judgement. The narrative flow was not stopped. Nevertheless, the interviewer’s prior knowledge of the patients could have influenced the interview. The interviewer’s gender could also have influenced the interview.

## Conclusion for practice

- Despite significant developments in recent years, endometriosis continues to be marginalised at times. Further resources need to be made available to improve research into the disease.
- It is possible to positively influence the experience of endometriosis patients with an app.
- Nocebo information can be avoided. Young patients, patients with a recent diagnosis or patients following rehabilitation may benefit more from the educational elements of an app.
- It is necessary to evaluate user behaviour after prescribing a DiGa.
- The adherence behaviour of patients can possibly be increased through a better understanding of the DiGa. An introduction or support at the start of use could be useful, similar to the prescription of medication.
- Patients stop using the app for various reasons, including concerns about time consumption, data protection, redundant information, and lack of effectiveness.
- Academic research without monetary interests must investigate DiGas.

## Supplementary information

**Additional file 1.**

**Additional file 1.**

## Data Availability

The data that support the findings of this study are not openly available due to reasons of sensitivity and are available from the corresponding author upon reasonable request.

## Abbreviations

*DiGa*: Digital health application
*I*: Interviewer
*B*: Interviewee
*SRQR*: Standards for Reporting Qualitative Research
*BfArM*: Federal Institute for Drugs and Medical Devices
