# Supplementary material for: Experiences of patients with endometriosis with a digital health application: a qualitative analysis
Source: Arch Gynecol Obstet. 2024 Jul 27;310(4):2253–63. doi: 10.1007/s00404-024-07651-7 (PMC11393288; doi:10.1007/s00404-024-07651-7)
Supplement: Supplementary file 1 — Additional file 1. [file 404_2024_7651_MOESM1_ESM.pdf]

Interview guide: Experiences of patients with endometriosis with a novel app-based, German-language digital health application (DiGa) - a qualitative analysis

Setting	Uniform declaration before the interview	Icebreaker
Pain outpatient clinic, university clinic, an assumption of strangeness cannot be credibly conveyed.<br><br>Goal: Create openness<br><br>Focused or semi-structured guided interview<br><br>Open questions first, four topics. Follow-up questions are possible in order to get to relevant aspects.<br><br>The interview always opens with the same explanations.<br><br>The aim is to produce text.<br><br>Narrative-generating questions, with few presuppositions.<br><br>Questions arranged in chronological order.	This interview is about your feelings, attitude and view of things.<br><br>Patients should tell their stories as freely as possible and are only given a rough direction.<br><br>There is no right or wrong. What is important is what YOU think. You will not be judged. Your statements are anonymous. Your statements have no influence on the therapy or are otherwise used outside the research.<br><br>We just need to be able to count on you to speak openly and truthfully.	**Demographic data:**<br><br>Age<br><br>Relationship status?<br><br>Place of residence (size)?<br><br>Level of education?<br><br>Annual income (< 50k, up to 100k, more)<br><br>Do you have children? How many?<br><br>Want to have children?

Leading question (narrative prompt)	Check - was this mentioned? Memo for follow-up questions - ask if not addressed by patients. Adapt wording	Specific questions - Ask in this formulation at the end	In-deep questions
Part 1:<br><br>Tell us about your medical history, from the beginning until today. In addition to the facts and figures, we are also interested in how you personally experienced the disease.	Pain:<br><br>• Social restrictions<br>• Movement<br>• Symptom control possible?<br><br>Emotions:<br><br>• Happy/sad<br><br>Social support<br><br>Self-image:<br><br>• Clothing, appearance, self-confidence<br><br>Workplace<br><br>Desire to have children<br><br>Sexual intercourse<br><br>Medical treatment<br><br>Coping strategies/information about the disease	• How did you find out about your illness? Describe exactly. How did you feel about it?	Can you tell us more about this?<br><br>What happened next?<br><br>What was that about...?

Leading question (narrative prompt)	Check - was this mentioned? Memo for follow-up questions - ask if not addressed by patients. Adapt wording.	Specific questions - Ask in this formulation at the end	In-deep questions
Part 2:<br><br>Tell us what it was like with the app? From the prescription to today and how you experienced it.	Functionality:<br><br>• Download<br>• Pay<br>• Use<br><br>Individual aspects:<br><br>• Eduction (information compared to previous information)<br>• Nutrition<br>• Relaxation<br>• Sport<br>• Medication - operations etc.<br><br>Usage behavior<br><br>• How has user behavior changed over time?	• Is enough/too little/too much being done to combat the disease?	Can you tell us more about this?<br><br>What happened next?<br><br>What was that about...?

Leading question (narrative prompt)	Check - was this mentioned? Memo for follow-up questions - ask if not addressed by patients. Adapt wording.	Specific questions - Ask in this formulation at the end	In-deep questions
Part 3:<br><br>Tell us what you want to improve in the future. How can an app support you in this?	Improvement app<br><br>Improvement therapy in general<br><br>Wishes and framing of the diagnosis	What are your thoughts on endometriosis and treatment?<br><br>(surgery cures, diet cures, yoga cures, nothing cures, what relieves? acceptance?)<br><br>What should be improved in the app?<br><br>Can you think of anything else that has not yet been mentioned?	Can you tell us more about this?<br><br>What happened next?<br><br>What was that about...?
