# Supplementary material for: Experiences of patients with endometriosis with a digital health application: a qualitative analysis
Source: Arch Gynecol Obstet. 2024 Jul 27;310(4):2253–63. doi: 10.1007/s00404-024-07651-7 (PMC11393288; doi:10.1007/s00404-024-07651-7)
Supplement: Supplementary file 2 — Additional file 1. [file 404_2024_7651_MOESM2_ESM.pdf]

List of codes

**List of codes**	**Frequency**
Code system	744
Factors influencing the experience of illness	0
Importance of conservative therapy	8
Importance of multimodal therapy	14
Significance of invasive intervention	28
Recognition of individuality of the disease	21
Promoting self-efficacy	9
Coping strategies	16
Association and framing of the disease	24
Information gathering	50
Internet	11
Literature	7

Social environment	3
Stakeholder healthcare system	14
Better information for doctors	15
Self-help groups	0
Social Media	6
Misinformation	1
Nocebo (ignorance)	14
Networking with those affected	20
Not alone with the disease	3
Course of the disease	10
Pain character	23
Diagnosis	18
Significance of a diagnosis - uncertainty	9
Importance of imaging - the image	5

Psychological impact	16
Desire to have children	24
Social dimension	23
Dependence	1
Social withdrawal	1
Support	15
Relativization/ Marginalization (+)	9
Pressure from outside	7
Presenteeism/absenteeism	2
„To be recognized“	23
Left alone	12
Helplessness of the helpers	4
Evaluation of the app	0
Contents of the app	0

Education	1
Pain diary	2
Physiotherapy	1
Relaxation exercises	2
Nutrition	3
Benefit in person	42
Information in the app	4
Functionality	28
Defects	37
Redundant education	7
Overload	5
Daily reminder of the disease	4
Usage behavior	26
Expectations of the app	4

Demographic data	15
Health insurance	13
Wishes for the future	26
Improvement of the app	2
Macro - systemic - political	11
Public relations	2
Assumption of costs	3
Research	6
Therapy	1
Diagnosis	3
Micro - individual	2
Flowers by the wayside	3
Quotable text passage	25
